# Life and bladder cancer: protocol for a longitudinal and cross-sectional patient-reported outcomes study of Yorkshire (UK) patients

**DOI:** 10.1136/bmjopen-2019-030850

**Published:** 2019-06-17

**Authors:** Samantha J Mason, Amy Downing, Penny Wright, Sarah E Bottomley, Andrew Winterbottom, Adam W Glaser, James W F Catto

**Affiliations:** 1 Leeds Institute of Cancer and Pathology, University of Leeds, Leeds, UK; 2 Academic Urology Unit, University of Sheffield Medical School, Sheffield, UK; 3 Fight Bladder Cancer, Oxford, UK

**Keywords:** bladder cancer, patient-reported outcome measures, health-related quality of life, outcome

## Abstract

**Introduction:**

Little is known about the impact of bladder cancer (BC) and its treatments on health-related quality of life (HRQL). To date, most work has been small in scale or restricted to subsets of patients. Life and bladder cancer is a cross-sectional and longitudinal study collecting patient-reported outcomes within two distinct cohorts.

**Methods and analysis:**

A longitudinal study will collect patient-reported outcomes at 3-monthly intervals from newly diagnosed patients. Eligible cases will be identified by recruiting hospitals and surveyed at baseline, 6, 9 and 12 months postdiagnosis to explore changes in outcomes over time. A separate cross-sectional cohort of patients diagnosed within the last 10 years across Yorkshire will be identified through cancer registration systems and surveyed once to explore longer-term HRQL in BC survivors. A comprehensive patient-reported outcome measure (PROM) has been developed using generic, cancer-specific and BC-specific instruments. The study will provide evidence about how useful these PROMs are in measuring BC patient HRQL. The outcome data will be linked with administrative health data (eg, treatment information from hospital data).

**Ethics and dissemination:**

The study has received the following approvals: Yorkshire and the Humber—South Yorkshire Research Ethics Committee (17/YH/0095), Health Research Authority Confidentiality Advisory Group (17/CAG/0054). Results will be made available to patients, funders, NHS Trusts, Clinical Commissioning Groups, Strategic Clinical Networks and other researchers.

**Strengths and limitations of this study**

- Patients with all stages and grades of bladder cancer (muscle invasive and non-muscle invasive) will be included in both studies.
- Cross-sectional survey data will be linked with available NHS data and longitudinal survey data will be linked with case report forms in order to optimise the levels of clinical and treatment information.
- Patient-reported outcome measures data will be used to understand differences between newly diagnosed patients, those undergoing treatment and those having completed treatment and compare different treatments and healthcare providers.
- Recruitment and retention of patients may be a challenge; particularly those from more deprived areas.

## Introduction

### Context

Bladder cancer (BC) is one of the most common human cancers and one of the most expensive to manage.^1–3^ Despite the cost of managing affected patients, BC receives a relatively low proportion of research and healthcare funding.

Most BCs are non-muscle invasive (NMIBC), which are managed by endoscopic resection with intravesical chemotherapy or immunotherapy and long-term surveillance.^4–6^ Around one-third of tumours are aggressive, muscle-invasive tumours (MIBC), requiring radical treatment.^7^ This radical treatment is usually radical cystectomy or radiotherapy, and includes treatment of adjacent viscera with regional lymph nodes, and often includes systemic chemotherapy. Information about which treatments are associated with greater survival rates is lacking.

Survival data for BC is around 50% at 5 years^8^. There are regional variations in service provision and outcomes, and patients with BC in Yorkshire have some of the lowest survival rates in the UK.^9^

### Patient-reported outcome measures

As cancer survival has increased in the UK, the quality of that survival has become increasingly important. Patients may experience short-term and long-term effects as a result of the cancer or cancer treatment, resulting in functional restrictions (eg, physical, emotional and social) or in specific symptoms (eg, urinary problems, fatigue, pain). These effects may impact on the everyday lives of individuals at home, at work, in relationships, recreationally and emotionally.

Patient-reported outcome measures (PROMs) seek to ascertain patients’ views of their symptoms, their functional status and health-related quality of life (HRQL).^10^ Studies using PROMs are being pursued in England to improve patient care by assisting clinicians to provide better and more patient-centred care, and providing data for evaluating practices and policies.^10^

### Policy

The collection and reporting of PROMs is a key priority as set out in the Government’s July 2010 White Paper, Equity and Excellence: Liberating the NHS, where the commitment was made to ‘extend PROMs across the NHS wherever practicable’.^11^ In order to improve understanding of the quality of life outcomes for cancer survivors, the National Cancer Survivorship Initiative developed a national survey of cancer survivors.^12^ Robust collection of PROMs was perceived as central to health service reforms in the UK and essential for the improvement of cancer outcomes through the provision of evidence for domains 2 and 3 of the new English health framework; enhancing HRQL of individuals with long-term conditions and enhancing recovery from ill-health, respectively.^13^ Hence, the development and delivery of evidence-based PROMs surveys was pivotal to current national health policy. The National Cancer PROMs programme was established and developed methodology for population-based PROMs surveys.^14^ Subsequent national roll-out included evaluation of all individuals’ postcolorectal cancer diagnosis.^15^ In addition to this, head and neck cancer PROMs research has recently been published^16 17^ and a current study is measuring PROMs in British prostate cancer patients, as part of a Prostate Cancer UK/Movember survey (life after prostate cancer diagnosis (LAPCD)).^18^

The life and bladder cancer (LABC) study will deliver evidence framed in terms of current health policy, thereby supporting health and social care organisations to recognise and develop programmes to address unmet needs and maximise HRQL for patients living with and beyond BC.

### Current knowledge

Literature suggests that two domains are of primary concern to BC patients: urinary and sexual.^19^ However, the same research stated that there are few HRQL studies in BC, due to a lack of standardised instruments of assessment.

In recent years, BC-specific PROMs have been developed. Many focus on a single type of BC or treatment type and few have been externally validated or surveyed large populations.^20–22^

A review of BC HRQL research stated that there are limitations in this field of work due to the methodology used in research, models used and the heterogeneity of disease clinical characteristics.^23^ As such, choosing appropriate PROMs to evaluate HRQL in BC patients is challenging for researchers and clinicians. The review recommended that future research should develop a comprehensive model of HRQL, that is, sensitive to changes in NMIBC and MIBC.

The majority of BC HRQL research focusses on MIBC patients and compares type of urinary diversion.^23^ Findings showed that regardless of the PROM used, the type of urinary diversion is not a consistent predictor of global HRQL but can impact on functionality, such as urinary function.^23 24^

Comparatively fewer studies have reported on the HRQL of patients with NMIBC. Longitudinal research in this area measured the HRQL of 244 NMIBC patients treated with transurethral resection of bladder tumour (TURBT), with or without intravesical therapy, over a year period.^25^ Over the year urinary function significantly improved, bowel function and sexual bother remained stable and sexual function decreased. Mental health was also found to be statistically lower in patients than the control population at baseline and 6 months, before improving at 12 months.^25^

Few large-scale studies of BC (NMIBC and MIBC) HRQL have been conducted, with research to date being cross-sectional. Research in the USA, of patient’s ≥65 years old found poorer physical functioning in patients with MIBC, persisting for >10 years postdiagnosis.^26^ European PROMs work with 823 German patients found that BC patients in both groups reported poorer emotional and physical functioning than in the general population.^27^ Both studies used generic PROMs or generic cancer PROMs. The lack of large-scale PROMs research in BC is detrimental as it hinders patient pathways and patterns of care, limits the accuracy of counselling and masks inequalities in care.

### Study aims

#### Primary aims

1. To describe the HRQL of patients living with BC diagnosed in Yorkshire and the Humber.
2. To gain a deeper understanding of the variation in outcomes.
3. To identify areas of unmet need.

#### Secondary aims

1. To develop a PROM tool to collect and interpret data from patients with BC diagnosed in Yorkshire and the Humber, with a view to extending into a national survey and ultimately improving clinical care.
2. To explore if and how HRQL is associated with or predicted by disease, treatment and/or patient characteristics in Yorkshire and the Humber, with a view to informing service delivery in order to better meet patient needs.
3. To use PROMs data to understand differences between newly diagnosed patients, those undergoing treatment and those having completed treatment for BC in Yorkshire and the Humber and to compare different treatments and healthcare providers.
4. To report PROMs results, allowing providers to see the responses from their patients and to identify any areas of concern.

The study will achieve these aims through 3 workstreams. The study will collect data from patients diagnosed at 15 sites across Yorkshire and the Humber providing BC care: Airedale, Barnsley, Bradford, Calderdale, Chesterfield, Doncaster, Harrogate, Hull and East Yorkshire, Leeds, Mid Yorkshire, North Lincolnshire and Goole, Rotherham, Sheffield, South Tees and York.

## Methods and analysis

The LABC study began in May 2016 and will run until 30 April 2020.

### Patient eligibility

Inclusion criteria for the longitudinal survey:

- A new diagnosis of BC with no previous history of the disease.
- No more than 3 months postdiagnosis at the time of identification. Date of diagnosis is taken as the date of the first tumour resection (TURBT) with histological confirmation of BC.
- Able to complete the survey form by themselves or with help.
- Able to read and understand English.

Inclusion criteria for the cross-sectional survey:

- A previous diagnosis of BC within the last 10 years. Date of diagnosis is taken as the date of the first tumour resection (TURBT) with histological confirmation of BC.
- Diagnosed by one of the NHS hospitals in Yorkshire and the Humber.
- Not taking part in the longitudinal study.
- Able to complete the survey form by themselves or with help.
  Exclusion criteria both surveys:
- Age under 18 years.
- Participants who are prisoners in the custody of HM Prison Service with an HMP address.
- Lack the capacity to give informed consent—this may be due, for example, to psychopathology, cognitive dysfunction or learning difficulties (longitudinal survey).
- Previous diagnosis of BC (longitudinal survey).
- Patients who register type 2 objections (cross-sectional survey).^28^

### Workstream 1: PROMs instrument refinement

A survey instrument has been developed that covers a range of generic and cancer-specific PROMs that address both NMIBC and MIBC populations. The survey will also contain items regarding treatments received, sociodemographic details, lifestyle questions and the patient perspective of their disease, treatment, needs and experiences. This content was informed by several factors. These include the incorporation of questionnaire measures used by colleagues in similar surveys and the experiences from these surveys (including response rates), the undertaking of a scoping and systematic review of PROMs used in BC research, availability of routine demographic and health data (to avoid duplicate collection of information), potential literacy issues experienced by patients from more deprived areas, questionnaire burden, item duplication/redundancy, costs and permission, and the priorities of different coapplicants and advisory group members, including service users were also considered.

#### Survey measures

##### Generic HRQL

The included measures are:

1. EuroQol (EQ-5D-5L): this measure records problems on five domains; mobility, self-care, usual activities, pain/discomfort and anxiety/depression.^29^
2. Short Warwick-Edinburgh Mental Well-Being Scale (SWEMWBS): a positive construct of emotional well-being. SWEMWBS is a UK validated shortened, 7-item version of the Warwick-Edinburgh Mental Well-Being Scale.^30^

##### Cancer-specific and BC-specific

These measures include:

1. European Organisation for Research and Treatment of Cancer core questionnaire (EORTC QLQ-C30): full questionnaire.^31^
2. BC-specific module consisting of merging the items of EORTC QLQ-BLM30 (MIBC module) and EORTC QLQ-NMIBC24 (NMIBC module)^21 32^ as they share a number of items, allowing for patients with both NMIBC and MIBC to be surveyed while reducing patient burden.
3. Social Difficulties Inventory (SDI): this measure was developed to assess everyday problems experienced by cancer patients.^33^ Sixteen of the items form three subscales: everyday living, money matters and self and others. These scales form a measure of social distress.^34^ As in previous research, three individual SDI items on difficulty with sexual matters (covered in detail elsewhere), housing and any other difficulty have been excluded due to poor endorsement in the pilot work of previous research.^18^
4. Bladder Utility Symptom Scale: a recently developed tool consisting of 10 questions and a visual analogue scale, designed for use with a variety of BC patients.^35^

##### Patient, clinical, lifestyle and sociodemographic characteristics

These include:

1. Treatment items informed by BC clinicians, patients, a BC charity support website and experts.
2. Comorbidity item (a list of possible conditions).
3. Standard sociodemographic items informed by the Office for National Statistics and other sources.
4. Support for previous mental health problems, taken from the National Comorbidity Survey.^36 37^
5. Item about carer status included in recognition of the increasing number of carers.
6. Godin Leisure-Time Exercise Questionnaire,^38^ comprising two items which assess exercise behaviours.
7. Three items about cigarette and e-cigarette use.
8. Two items about employment status relating to employment status at diagnosis (baseline longitudinal survey only), current employment status and sick pay received.

##### Patient perspective measures

The included measures are:

1. Decision Regret Scale^39^ which provides an indication of healthcare postdecision regret at a set moment in time.

Cognitive testing of the survey has been carried out by the approved survey provider (Quality Health) with a group of BC patients. Appropriate revisions were made to the surveys.

A summary of the questionnaires included in each survey is included in table 1.

**Table 1:** Overview of questionnaires included in cross-sectional and longitudinal surveys

Domains	Questionnaires/items	Time points			
		T1/cross-sectional	T2	T3	T4
Your overall health	EQ-5D-5L	Yes	Yes	Yes	Yes
Your treatment	Treatment items	Yes	Yes	Yes	Yes
	Decision Regret Scale	No	No	No	Yes
How things are for you now	EORTC QLQ-C30	Yes	Yes	No	Yes
	EORTC merged bladder cancer modules (NMIBC24 and BLM30)	Yes	Yes	No	Yes
	Bladder Utility Symptom Scale	No	No	Yes	No
Your everyday life	Social Difficulties Inventory	Yes	Yes	No	Yes
Your care needs	Supportive Care Needs Survey 34	No	No	Yes	No
Your emotional well-being	Short Warwick-Edinburgh Mental Well-being Scale	Yes	Yes	No	Yes
Your exercise habits	Godin-Leisure-Time Exercise Questionnaire (prior to diagnosis)	Yes	No	No	No
	Godin-Leisure-Time Exercise Questionnaire (current)	No	No	No	Yes
Smoking	Cigarette smoking	Yes	No	No	Yes
	E-cigarette smoking	Yes	No	No	Yes
	Passive smoking	Yes	No	No	Yes
About you	Age	Yes	No	No	No
	Marital status	Yes	No	No	Yes
	Ethnicity	Yes	No	No	No
	Other conditions (comorbidities)	Yes	Yes	Yes	Yes
	Height	Yes	No	No	No
	Weight	Yes	Yes	Yes	Yes
	Support for mental health or alcohol/drugs	Yes	No	No	Yes
	Carer	Yes	No	No	No
Your employment status	Employment (prior to diagnosis)	Yes	No	No	No
	Employment (current)	Yes	Yes	Yes	Yes
	Sick leave and sick pay	Yes	Yes	Yes	Yes
Total number of questions		114	101	56	108

EORTC QLQ-C30, European Organisation for Research and Treatment of Cancer Core questionnaire.

### Workstream 2: longitudinal PROMs survey

#### Sample strategy and size

National Cancer Registration and Analysis Service (NCRAS) data suggest that there were 1902 new cases of BC diagnosed in Yorkshire and the Humber in 2014 and that at 1 year postdiagnosis 1283 patients were still alive (table 2).

**Table 2:** Estimated number of newly diagnosed bladder cancer patients and survival 1 year postdiagnosis by study site

Sites	No of patients diagnosed	No alive at 1 year
South Tees	46	31
Leeds	228	153
York	204	138
Airedale	89	60
Harrogate	79	54
Calderdale	128	86
Bradford	76	52
Hull and East Yorkshire	208	141
North Lincolnshire and Goole	156	105
Mid Yorkshire	200	134
Sheffield	188	127
Doncaster	159	107
Rotherham	92	62
Barnsley	39	25
Chesterfield	10	7
Total	1902	1283

#### Recruitment

Local NHS clinical research teams, at the participating research sites, will identify potential patients following discussion in routine BC multidisciplinary team (MDT) meetings and confirm that the patient can be approached to take part in the LABC study. After checking the patient is eligible, eligible patients will be approached by a member of the clinical research team about the study. Patients will be given study information and will be asked to sign a consent form giving informed consent if they wish to take part. Patients giving informed consent in person will be asked to complete a participant details form.

The participant details form will ask patients to complete their contact details and preferred method for completing the survey (online, on paper or over the telephone). One copy of the signed consent form/participant details form will be filed by the clinical team. Patients will have a copy to keep.

Patients who give informed consent to take part will be surveyed at baseline (<3 months after diagnosis) and again at 6, 9 and 12 months postdiagnosis.

Patients who could not be approached in person will be sent a letter from their MDT lead clinician, inviting them to take part. Study documents and two copies of a sheet comprising the consent form and participant details form will be included with a freepost return envelope. The signed consent form/participant details form will be returned to the clinical team.

If no consent form has been received within 2 weeks, then a member of the clinical research team will contact potential participants. The mode of contact will be by either telephone or letter, at the discretion of the clinical research team.

Information from consenting participants will be sent from the clinical sites to Public Health England (PHE) by secure NHS email. PHE will store this information and forward the information to Quality Health.

**Figure 1:**
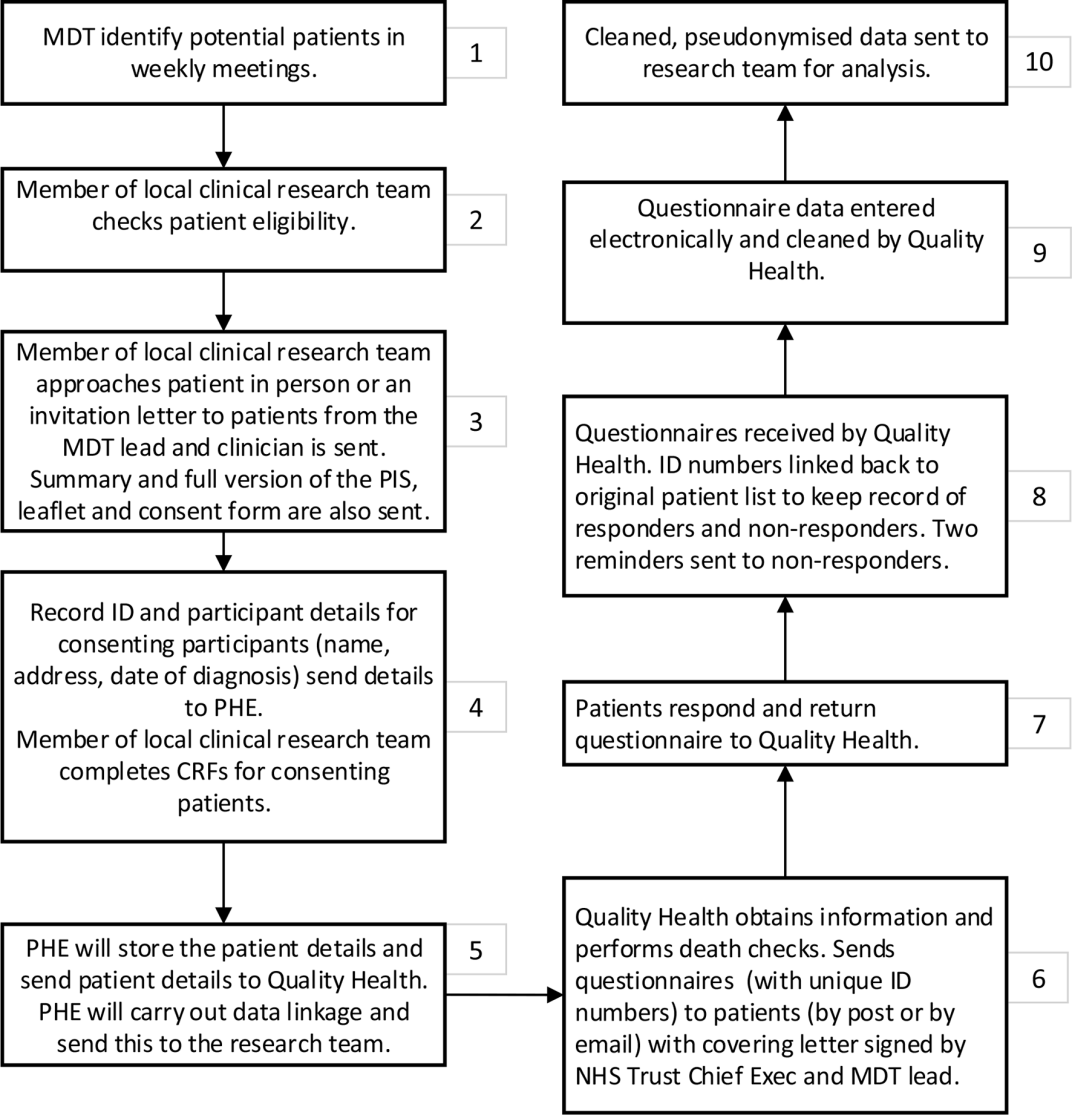
Study data flow for the longitudinal study (workstream 2). NCRAS, National Cancer Registration and Analysis.

Quality Health (acting under an agreed data processor contract) will send the data to NHS Digital who will carry out a list clean of data to check the vital status of patients, identify any patients who have died and provide updated address checks for participants requesting posted questionnaires. Once this has been carried out, NHS Digital will send the data back to Quality Health who will send out the surveys. Two reminders will be sent to non-responders (with additional death checks performed). Details of the methodology for workstream 2 is summarised in table 3 and figure 1.

**Table 3:** Overview of study methodology for longitudinal and cross-sectional cohorts

	Longitudinal cohort	Cross-sectional cohort
Data source	NHS Trusts	Cancer registry
Confirmation of diagnosis and eligibility	MDT leads at Trusts and local research teams at each NHS Trust	Bladder MDT lead
Exclusions	<18 years; lack capacity to give informed consent; previous diagnosis of bladder cancer; participants who are prisoners in the custody of HM Prison Service with an HMP address	<18 years; registered a type 2 objection; participants who are prisoners in the custody of HM Prison Service with an HMP address
Death checks	NHS Digital	NHS Digital
Type 2 opt out checks	N/A	NHS Digital
Survey mail-out	Quality Health	Quality Health
Language	English	English
Survey dates	Starts April 2019	Autumn 2018 to Spring 2019
Estimated potential no of patients	1902	4000

**Figure 2:**
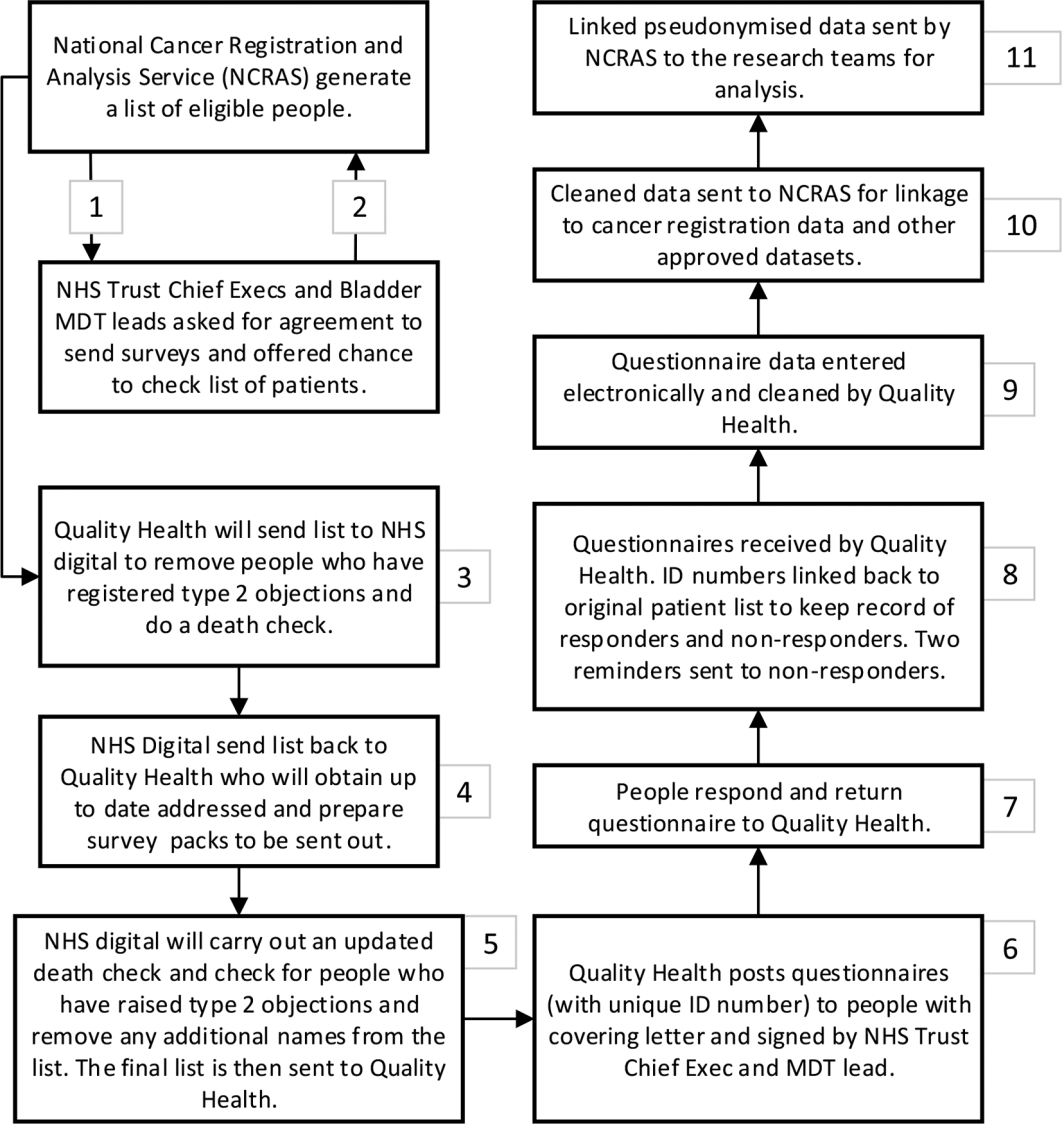
Study data flow for the cross-sectional study (workstream 3). CRFs, case report forms; PHE, Public Health England; PIS, patient information sheet.

#### Case report forms

Case report forms (CRFs) will be completed at each recruiting centre, for patients who consent to take part in the longitudinal study at baseline and 12 months postdiagnosis. These CRF’s will be sent securely to the project coordinator at the University of Sheffield using nhs.net email. These CRFs will be used to understand current diagnosis and treatments and any scheduled future treatments. This will allow for more in-depth interpretation and understanding of the quality of life data gained from the surveys. The CRFs will be linked to the survey responses using the study identification (ID) assigned to the patient.

### Workstream 3: cross-sectional PROMs survey

#### Sample strategy and size

Data from NCRAS suggest there are an estimated 7000 patients alive in Yorkshire and the Humber with a history of BC, including 20% with a >10-year history. We estimate that after death checks and exclusions due to type 2 objections there will be an estimated 4000 people, who were diagnosed with BC up to 10 years ago, eligible to take part in the cross-sectional study.

#### Recruitment

The methodology follows that used by the National Colorectal PROMs survey, England 2013^14^ and the LAPCD study.^18^ The study team will write to the chief executive and BC MDT lead of each Trust to seek their permission to survey people treated by their trust in the last 10 years. Trusts will be invited to verify the list of identified patients and filter any patients where contact would be inappropriate. For the Trusts that agree to take part, NCRAS will extract a list of eligible patients and send this to NHS Digital for up-to-date address tracing and death checks. Once these checks have been completed, the information will be passed on to the approved survey provider, Quality Health. Details of the methodology for workstream 3 is summarised in table 3 and figure 2.

The survey will be sent out with a covering letter from the treating NHS Trust’s Chief Executive and MDT lead and a summary and full version of the participant information sheet. The covering letter will indicate that the survey is only to be completed if the patient has received a diagnosis of BC.

Patients who agree to participate will complete and return the survey in prepaid freepost envelopes to Quality Health. Instructions will be provided to allow patients to complete online or over the phone if they wish.

As in the longitudinal survey, no identifying information will be included in the surveys but a unique reference number (URN) will be used to keep track of which patients have returned the survey or who have opted out. Two reminders will be sent (with additional death checks performed).

### PROMs completion: written or electronic options

For the longitudinal study, participants will be asked how they wish to be contacted, as part of the consent process. The options include written (letters), electronic (email address) or by telephone (verbal, from Quality Health).

Participants will be allowed to change between modes of communication and completion at their discretion. Participants who want to change their method of completion will be instructed to contact the study helpline, who will facilitate this. The survey will be sent out either electronically or by post (dependent on which option the participant identified as their preferred way to complete) with a covering letter from the study principal investigators.

People eligible to take part in the cross-sectional survey will be a sent a survey pack through the post. The survey pack will include a paper copy of the survey, a freepost envelope for returning the paper survey, a summary patient information sheet (PIS), a full version of the PIS (so that they have more details about the study), a covering letter and information and instructions for accessing the survey online (eg, their unique username and password, website address). Participants will be asked to complete and return the survey either by post in a provided freepost envelope, by completing the survey online or over the telephone. By completing the survey, the participant is consenting to take part in the study.

Paper versions of the survey will include a copy of the survey and a freepost envelope in which to return the survey to Quality Health. Details of how to complete the survey over the telephone will be included in the covering letter.

For the online option, a web address (URL) will be provided that takes the person to the survey. This information will enable them to access the survey online. Participants can complete the survey online from any internet accessible location, at any time. Completion of the online survey will take ~30 minutes, although participants can spread-out completion (eg, over a number of days) if they wish.

The surveys will not contain any personal information (ie, no names or addresses) but will be assigned a URN. The URN can be linked back to the consented patients to keep track of which participants have returned the survey or have opted out (by returning the survey blank or phoning the dedicated survey helpline).

Quality Health will send two reminders (with additional checks performed each time) to participants taking part in the studies, who do not complete the survey. The first reminder will be a letter and the second reminder will be a full invitation pack.

Quality Health will scan and input data for the completed surveys and clean the data, including removing any identifying information that patients may have provided. The cleaned, electronic dataset of pseudonymised survey responses will be sent, alongside a study ID number, to the research team for analysis.

### Freephone helpline

For both the longitudinal and cross-sectional surveys, a 24-hour freephone service will be provided during periods when the surveys are live. Any queries pertaining to BC symptoms or disease management will be directed to the Fight Bladder Cancer charity helpline service. Fight Bladder Cancer are a UK-based BC charity founded and run by BC survivors and families to support people impacted by BC, raise awareness of the disease, support research and campaign for changes in policy.

**Figure 3:**
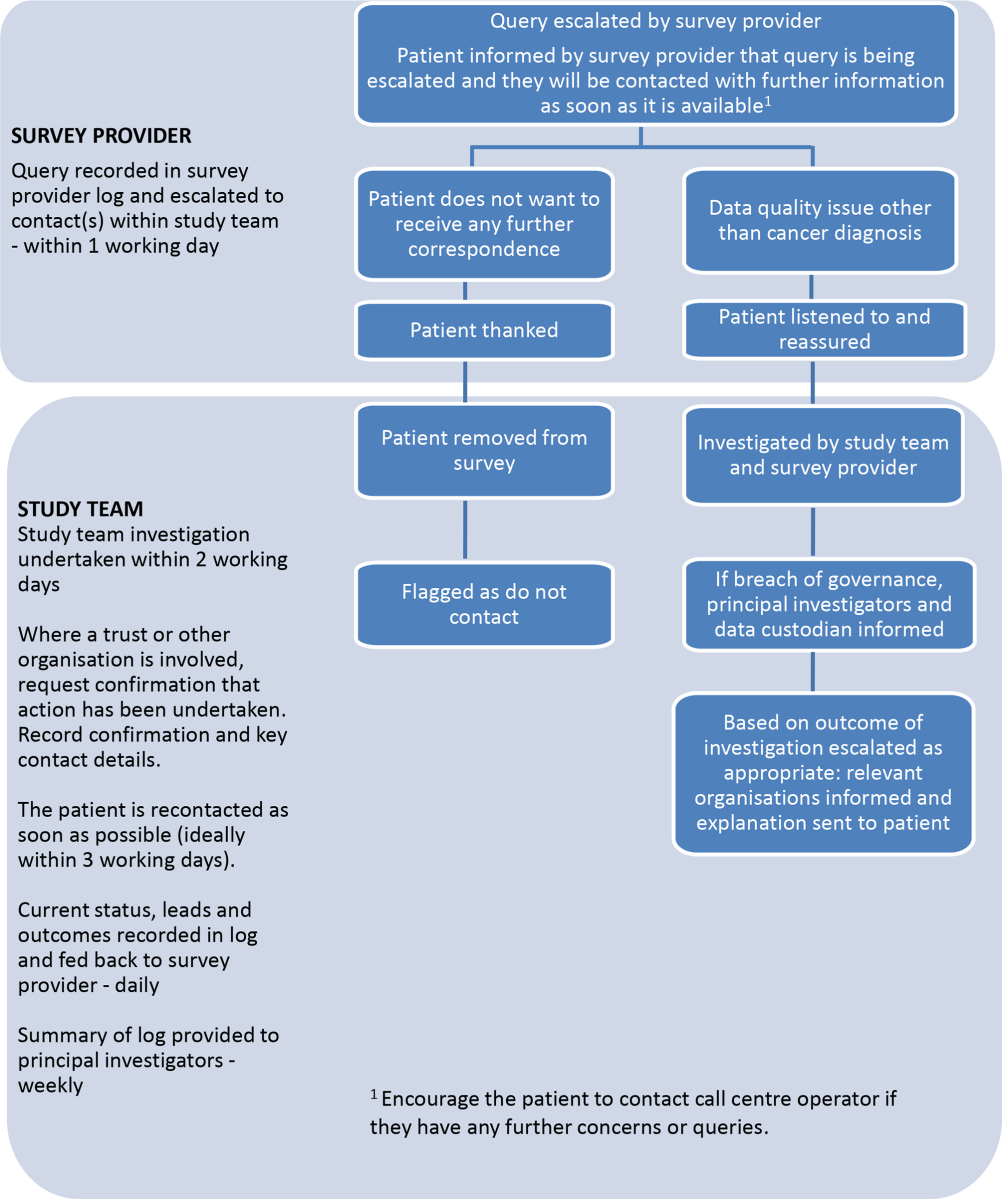
Patient query escalation policy for the longitudinal study (workstream 2).

**Figure 4:**
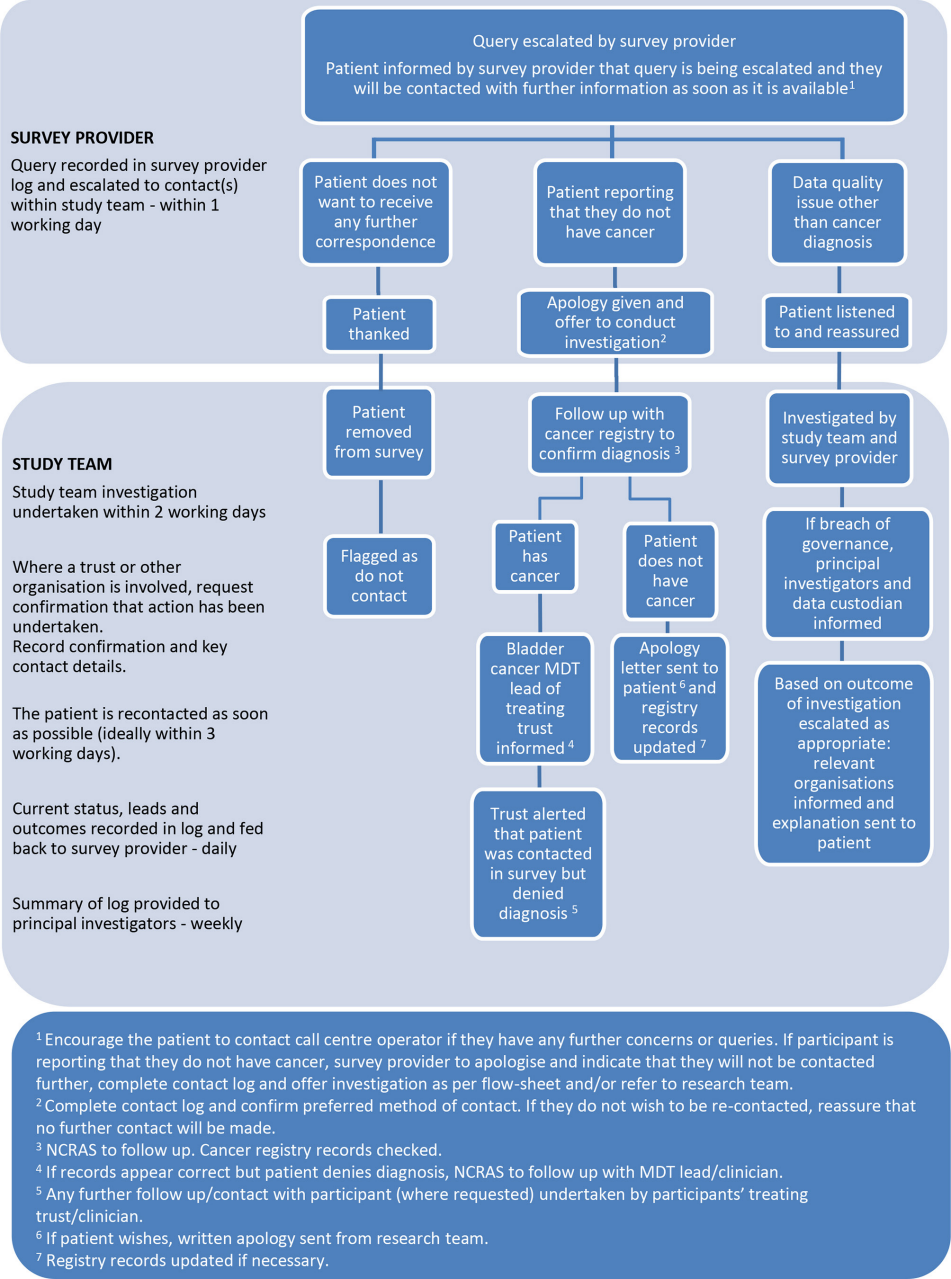
Patient query escalation policy for the cross-sectional study (workstream 3). NCRAS, National Cancer Registration and Analysis Service.

For other enquiries, for example, where the patient informs the helpline that they do not have cancer or that they do not wish to be contacted again, escalation processes have been developed (figures 3 and 4). Procedures to rapidly manage and report any incidents arising from the survey have been established. It is not possible to foresee all possible queries or issues that will arise during the study duration. However, processes have been developed to deal with issues that have arisen in previous PROMs surveys.

### Data linkage

The survey response data from the cross-sectional study will be sent to NCRAS where it will be linked to a number of routine data sets in order to maximise the amount of clinical and treatment information available. The datasets for linkage include:

- *Cancer registration*: these data will provide staging information, area-level deprivation, confirmation of reported treatments and validation of age, sex and ethnicity. Cancer registration data are available for all BC patients and can be used to assess responder bias (comparison of the respondents and non-respondents in terms of age, deprivation and so on).
- *Hospital admissions*: these data will provide information on inpatient and outpatient admissions, including treatments received, hospital visited, specialty, length of stay and other conditions/comorbidities.
- *Radiotherapy*: these data can provide information on type of radiotherapy (long or short course), number of fractions, intent and so on.
- *Chemotherapy*: the systemic anticancer therapy dataset can provide information on inpatient, day case, outpatient and community setting chemotherapy treatment.

All linkage will be undertaken by trained staff at NCRAS who have approvals to work with identifiable data. Linkage will be approved by the Office for Data Release. Once linked, the data will be pseudonymised (names, addresses, dates of birth, NHS numbers removed) and securely transferred to the study team for analysis.

### Data analysis and reporting

Descriptive statistics will be used to report the survey results and describe the health outcomes of people with BC who have a variety of stages, grades and treatments. The outcome variables, that is, EQ-5D-5L, EORTC QLQ-C30, the merged EORTC BC modules (EORTC QLQ-NMIBC24 and EORTC QLQ-BLM30) and SDI, will be analysed according to stage/severity of disease, treatment type, comorbidity, age, ethnic and sociodemographic group (and other relevant variables). These descriptive analyses will identify potential relationships of interest which can be further investigated.

If sufficient data are collected, regression modelling will be used to investigate associations among the different variables and to identify statistically and clinically significant risk factors and predictors of health outcomes. In order to be robust, analyses will require appropriate adjustment for case mix and other confounding factors.

The longitudinal study will allow measurement of any changes in outcomes over time. For example, differences in EQ-5D scores could be calculated between the time points and this would allow assessment of whether outcomes improve, decline or remain static. Interpretation is difficult, however, as there is no information regarding the individuals’ health before their cancer diagnosis. Normative data from the general population will be used, where these are available, in order to compare the health of people with BC with those in the general population and to assess whether their health returns to a ‘normal’ level over time.

New instrument development is not being undertaken as part of this work. However, there is the opportunity to explore and check the psychometric properties of EORTC QLQ-C30 as a full psychometric evaluation of this PROM has not been undertaken with BC patients. There is also the opportunity to explore and check the psychometric properties of newer, less well-established questionnaires, particularly the merged EORTC BC module questions and to determine the most fitting instruments for future BC PROMs work.

### Management and oversight

An advisory group will be used to provide expert knowledge for study design, interpretation, analysis and reporting. The group comprises two service user members plus a number of (1) health professionals (2) researchers, with commitment to, and detailed expertise, research knowledge and experience of user concerns and priorities. The advisory group and project team will meet every 6 months.

The principal investigators, project manager and other relevant team members will have weekly telephone meeting to review progress.

### Patient and public involvement

A patient representative was involved in the grant proposal, research design and choice of outcomes. Two patients are members of the study advisory group, contributing as lay advisors to all aspects of this research project. Before finalising the surveys, Quality Health carried out cognitive testing of the surveys with patients.

## Ethics and dissemination

It is intended that the study will provide detailed information about the HRQL of BC patients. These data will be used to influence and drive service improvements, provide information to both patients and clinical teams which will allow them to make informed and appropriate treatment decisions, to understand the needs of different groups of BC patients and encourage service providers to have measures in place to pre-empt and support those needs should current and future patients have them (optimise the provision of support pre-treatment and post-treatment) and inform future research. The study will also go some way to providing the research with an understanding of how useful the PROMs used in the study detect changes in HRQL of BC patients and whether they should be employed in future research.

The success of this study relies on correctly identifying, consenting and contacting eligible patients without causing undue distress, and obtaining a high response rate from a representative sample of BC newly diagnosed patients and survivors. The study results must be disseminated widely and effectively to provide maximum impact.

### General data protection regulation

There were delays in obtaining all relevant approvals for workstreams 2 and 3 due to regulatory changes in the European Union (including the UK), where the standard for data protection is now the general data protection regulation (GDPR) rather than the Data Protection Act (1998).

Studies collecting data after 25 May 2018 are required to make their research GDPR compliant. GDPR is a regulation on data protection and privacy for all individuals in the European Union. Regulations require organisations that are involved in data processing to be lawful, fair and transparent when processing or controlling the processing of personal data. GDPR requires each activity of processing data to have a legal basis under this legislation. For universities, NHS organisations, research council institutes or other public authority, the processing of personal data for research should be a ‘task in the public interest’. Under GDPR, consent is no longer considered the legal basis for processing data.^40^

It is a requirement of NCRAS/PHE and NHS Digital that studies using their data are GDPR compliant. Consequently relevant amendments and additions were made to all study documents and published study information before workstreams 2 and 3 could be approved. Amendments pertained to how study data would be processed and used by all organisations involved in the research. The research methodology was not changed as part of these amendments. This process may be refined as part of future research.

### Ethical and safety considerations

The methodology for the longitudinal survey requires patients to give their informed consent to take part, and explains how patients can withdraw from the study. As patients will be recruited as a result of receiving a diagnosis of BC and will receive information about the study and what will happen if they consent to taking part, there should be minimal risk of patients becoming upset or angry at being contacted.

The methodology for the cross-sectional survey will follow that adopted in previous surveys (LAPCD,^18^ colorectal^15^) where the number of adverse events/symptoms was very low. In addition, approval will be sought from the treating Trust/MDT and they will be offered the chance to check the list of eligible patients and patients will be asked to give their informed consent to take part, in adherence with GDPR.^41^ The invitation letter that accompanies the survey will inform patients that if they did not receive a diagnosis of BC following investigations and/or treatment related to their bladder, to return all the enclosed documents, using the freepost envelope provided and will not be contacted again.

In both the longitudinal and cross-sectional studies, death checks will be carried out on a monthly basis and these data will be checked prior to survey mail-out; however, it must be acknowledged that even with the most stringent checks, a small number of individuals may have died very close to the time of survey mailing and will receive a survey. Despite all of these measures, it is not possible to predict the reaction of patients who receive a survey, for example, whether patients receiving a cross-sectional survey will become angry or upset at being contacted. The information accompanying the survey has been carefully worded, in a way similar to the wording of documents used in the LAPCD study and has been checked with the ethics committee in order to optimise positive reactions. In order to deal with any adverse events/symptoms, a procedure for rapid and timely response to, and support of, affected individuals has been developed.

### Patients who have raised a type 2 objection

From 29 April 2016, the Secretary of State for Health gave directions that required NHS Digital to establish and uphold type 2 objection requests expressed by patients. A type 2 objection is a request made by a patient. This is lodged with a general practitioner (GP) practice and indicates that personal identifiable information relating to the patient must not be disseminated or published for purposes beyond their direct care.^42^ In the cross-sectional study, a subsection of the patients identified by NCRAS may have raised a type 2 objection. Prior to mail-out, NHS Digital will identify and remove all patients who have registered a type 2 objection.

### Maximising response rates

Methods will be employed to promote as high of a response rate as possible, including sending two reminder letters, which has been shown to increase response rates, and the option to complete the survey over the phone as it is thought that BC patients in deprived areas have a lower literacy rate.^43^ It is known from previous PROMs surveys that there tend to be differences in the characteristics of those who do and do not respond, with the elderly, ethnic minorities and those from more socioeconomically deprived areas being less likely to participate. However, particularly for the longitudinal survey we expect some patients to drop out of the study over the 12-month period.

If, after using the methods above, there are differences between the responders and non-responders, statistical techniques can be used to adjust for variation in participation rates.

The use of online surveys will be explored during both studies. Response rates will be examined for variation by age, and other sociodemographic factors, and to see whether response rates can be increased using online methods.

### Dissemination plan

Dissemination of study findings will be undertaken through a series of reports, academic papers (open-access) and conference presentations. Additionally, all findings will be available on the dedicated study website. These outputs will provide understanding of key clinical, sociodemographic, psychosocial and service/organisational factors that have an impact on the generic and cancer-specific HRQL of BC patients and will also identify common issues where support, interventions or additional services are required for different groups of BC patients. An electronic report and toolkit will be available to key stakeholders to provide detailed anonymised information. The toolkit will enable each NHS Trust, Clinical Commissioning Group and Strategic Clinical Network to visualise the results for their organisation and to compare them against the national ‘average’. The study will also test the use of generic, cancer-specific and BC-specific PROMs with this patient group and provide feedback about how useful these tools are in measuring BC patient HRQL.

### Participant anonymity

Publications/reports on the study findings will make no reference to the identities of the patients who participated. When describing the clinical and sociodemographic characteristics of the sample, care will be taken to ensure that, if any values are small numbers, for instance, this information does not allow individuals to be identified. Access to medical records (outside of those who would ordinarily have access) will only be undertaken with patients’ explicit consent. All study data will be stored securely and accessed only by members of the research team on a strict need-to-know basis.

### Data storage and security

All electronic data transfers will be carried out using secure mechanisms, with appropriate encryption and use of randomly generated strong passwords. Data will be stored on secure networks and accessed only by members of the research team using discreet passwords. All members of staff involved in the study follow the relevant codes of practice concerning confidentiality, information security management and records management. Policies on safeguarding data will be adhered to.

For the period of the study, the pseudonymised longitudinal and cross-sectional survey data will be stored in a secure environment at the University of Leeds. The data will be accessed by approved members of the research team who will adhere to the agreed data security protocol and follow the relevant codes of practice concerning confidentiality, information security and records management.

Identifiable data for both workstreams will go to NCRAS and they will be the key holders for these data. NCRAS will hold the identifiable data for 10 years after the study closes. This would allow a clinical review of the initial findings to be done, subject to ethical approval and future funding, if the study were extended to other areas.

A 10-year data retention policy will be adopted for (1) hard-copy data (survey responses) and (2) electronic records (held by Quality Health). At the end of the study (24 months after the date of the first letter), the names and addresses used for the survey mail-out will be destroyed and the records retained for longer will be identified only by an ID number (with only NCRAS able to identify participants). Quality Health is an approved NHS provider and has met all the relevant information governance and security. Electronic copies will be held on secure networks and accessed only by approved members of the team. At the end of the study, the electronic survey data will be transferred to the Cancer Services Outcomes Dataset within NCRAS. This will be held according to the information governance arrangements that apply to all other datasets held by NCRAS (Section 251 approval to hold information on cancer patients).

## Supplementary Material

Reviewer comments
