# Correction: *Life and bladder cancer: protocol for a longitudinal and cross-sectional patient-reported outcomes study of Yorkshire (UK) patients*

**DOI:** 10.1136/bmjopen-2019-030850corr1

**Published:** 2019-07-09

Mason SJ, Downing A, Wright P, *et al*. Life and bladder cancer: protocol for a longitudinal and cross-sectional patient-reported outcomes study of Yorkshire (UK) patients. *BMJ Open* 2019;9:e030850. doi: 10.1136/bmjopen-2019-030850.

This article was previously published with an error in figures.

The correct figures are below:

Figure 1: Study data flow for the longitudinal study (workstream 2). NCRAS, National Cancer Registration and Analysis.

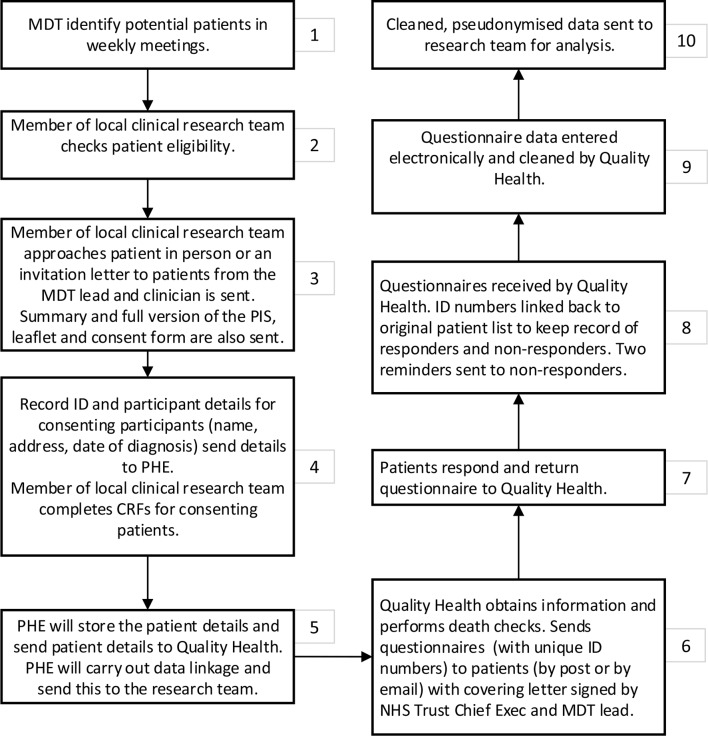



Figure 2: Study data flow for the cross-sectional study (workstream 3). CRFs, case report forms; PHE, Public Health England; PIS, patient information sheet.



