# Supplementary material for: Life and bladder cancer: protocol for a longitudinal and cross-sectional patient-reported outcomes study of Yorkshire (UK) patients
Source: BMJ Open. 2019 Jun 17;9(6):e030850. doi: 10.1136/bmjopen-2019-030850 (PMC6589036; doi:10.1136/bmjopen-2019-030850)
Supplement: Reviewer comments [file bmjopen-2019-030850.reviewer_comments.pdf]

PEER REVIEW HISTORY

BMJ Open publishes all reviews undertaken for accepted manuscripts. Reviewers are asked to complete a checklist review form (**http://bmjopen.bmj.com/site/about/resources/checklist.pdf**) and are provided with free text boxes to elaborate on their assessment. These free text comments are reproduced below.

ARTICLE DETAILS

**TITLE (PROVISIONAL)**	Life and Bladder Cancer: Protocol for a longitudinal and cross-sectional Patient Reported Outcomes study of Yorkshire (UK) Patients.
**AUTHORS**	Mason, Samantha; Downing, A; Wright, Penny; Bottomley, Sarah; Winterbottom, Andrew; Glaser, Adam; Catto, James

This article was not externally reviewed at BMJ Open. Protocols that have been independently assessed prior to submission to BMJ Open are usually fast-tracked to publication on the grounds that further substantial changes will not be possible. This independent assessment will usually be external, independent review for both a substantial grant award from a non-commercial or government funder and ethics approval.
